# Using a protection motivation theory framework to reduce vaping intention and behaviour in Canadian university students who regularely vape: A randomized controlled trial

**DOI:** 10.1177/13591053221144977

**Published:** 2023-01-12

**Authors:** Babac Salmani, Harry Prapavessis

**Affiliations:** 1 Western University, Canada

**Keywords:** behavior, intention, protection motivation theory, threat appraisal, vaping

## Abstract

Using Protection Motivation Theory (PMT), we examined the effect of threat appraisal information (perceived vulnerability-PV and perceived severity-PS) to reduce vaping intentions, and in turn reduce vaping use. Canadian university students (*n* = 77) who vape regularly were randomized to either PMT or attention control treatment conditions. Data were collected at baseline and 3 time points after the intervention: Day 7, Day 30, and Day 45. Participants assigned to the PMT group showed significant increases in PV, PS, and intentions to vape less (*p* ⩽ 0.05) compared to those in the attention control group. Less convincing evidence was found between treatment groups for vaping use. PS and PV predicted vaping intentions, whereas vaping intentions did not predict vaping use. It is suggested through this study that the threat appraisal components of PMT can be successfully manipulated to reduce the intentions to vape and to a lesser extent reduce vaping use among University vapers.

## Introduction

Canada has one of the highest rates of vaping usage in the world, with more than one-third of Canadian students having tried vaping products at some point in their lives, the highest rates of vaping being among young adults (18–24 years) (Statistics Canada, 2020). Evidence exists that although vaping has shown to assist with smoking cessation in adult tobacco smokers (Statistics Canada, 2020), long-term exposure to e-cigarette vaping may lead to nicotine dependence and increases in respiratory and cardiovascular health concerns (Herbert et al., 2014). Modern e-cigarettes produce several dangerous chemicals including acetaldehyde, acrolein, and formaldehyde. These aldehydes are known chemicals leading to possible lung disease, as well as cardiovascular disease (Ogunwale et al., 2017). E-cigarettes also contain a herbicide, acrolein, primarily used to kill weeds and may potentially lead to acute lung injury, chronic obstructive pulmonary disease (COPD), asthma, and lung cancer (Bein and Leikauf, 2011). In a study following a large population (*N* = 21,000) over a period of 5 years starting in 2013, comparing the development of chronic respiratory disease between people who vaped and those who never used e-cigarettes, those who vaped were 30% more likely to develop asthma and 60% more likely to develop COPD (Xie et al., 2020). More recently, in a study examining the effects of vaping on lung inflammation and injury, a strong association between vaping and acute lung injuries was revealed, likely involving cytotoxicity and neutrophilic inflammation caused by inhaled chemicals (Park et al., 2022). It has also been recognized that in humans, long-term use is not the only determining factor for adverse health events, even an isolated 5 minutes of vaping can cause changes in the way the lungs work and can lead to increased inflammation, risk of lipoid pneumonia (Gay et al., 2020) and cause a spontaneous pneumothorax (Skertich et al., 2019).

The existence of health risks associated with the use of e-cigarettes is robust, however, there is limited evidence to support the presence of health benefits and extent of health outcomes related to vaping cessation (Hammond et al., 2019). In a recent cross-sectional survey examining vaping-related adverse health events among daily vapers, former users self-reported greater adverse health events after cessation compared to current vapers. Moreover, users’ characteristics, usage status, and type of device and e-liquid used were found to be strong determinants of health, however, further examination is required (Pénzes et al., 2021). Similar to tobacco and cigarettes in the past, it may take several decades to fully understand the harm of using these vaping products and how reducing their use affects one’s health. Contemporary evidence suggests e-cigarette use may follow the trend of tobacco cigarettes. That being multiple long-term health risks with continued use (Callahan-Lyon, 2014). As the literature on the short-term health consequences of vaping behaviour continues to mount (Statistics Canada, 2020) and as the vaping market continues to grow in North America (Peruzzi et al., 2020), research identifying effective health behaviour change strategies to curve intentions to vape and vaping use are becoming increasingly paramount (Olmedo et al., 2018).

An important practical intervention question is will information about the protective health risks of vaping behaviour have any impact on vaping intentions and behaviour for self-identified, regular vapers? Rogers (1975) Protection Motivation Theory (PMT) offers a useful framework to study this intervention question. Broadly speaking, PMT is a major health psychology framework that explains the cognitive mediational process of behavioral change in terms of threat and coping appraisal. It is hypothesized through PMT that the motivation (intention) to protect oneself from danger is a function of four cognitive threat beliefs: (i) the perceived severity of the threat-PS, (ii) the perceived vulnerability of the threat-PV, (iii) the effectiveness of the coping response in averting the threat-RE, and (iv) the self-efficacious belief to perform the coping response-SE. Protection motivation (intention) is the proximal determinant of the protective health behavior. Hence, the cognitive threat beliefs (PS, PV, RE, and SE) should directly influence intentions, which in turn mediates its influence on behavior. Previous tobacco control studies provide evidence for the utility of PMT in changing cigarette smoking intentions and behavior (Lin and Chang, 2021; Smerecnik et al., 2011; Thrul et al., 2013; Wright et al., 2008; Yan et al., 2014). These findings, however, cannot be generalized to regular vapers, garnering further investigation of vaping behaviour instead of cigarettes.

### Current study

The purpose of the proposed study is to examine the effectiveness of an intervention grounded in the threat appraisal components of a PMT framework (i.e., PV and PS) that seeks to mitigate vaping intention and behaviour among Canadian university students that identify as regular vapers, vaping at least three times over 30 days prior to enrolling in the study (Gambaryan, 2018). Given the short existence of modern vaping devices, there is no research to support the health benefits of reducing or abstaining from vaping behaviour and the efficacious beliefs to engage in these behaviours (PMT coping appraisal components of RE and SE). Therefore, to maintain integrity in our intervention design, we formatted our study to include only the literature supporting health risk information (threat appraisal) of continued vaping. Regular vapers were targeted because theorists have suggested a stage-matched intervention approach to smoking health behaviour change (Prochaska and Goldstein, 1991). We steered towards young vapors as recent national statistics have found that the prevalence of current e-cigarette use is highest among Canadian adult youth between the ages of 16 and 24. More than half (53.1%) having tried vaping at some point in their lives and 15% of young adults, aged 20–24, are current e-cigarette users.

Hence, our research question was as follows: “Is health threatening information effective in motivating Canadian university students who regularly vape to reduce their intentions to vape and overall vaping behaviour?” Within this general research question, the following specific hypotheses were generated: (a) those exposed to the threat appraisal information grounded in the PMT components of severity and vulnerability will score higher on purpose-built questions reflecting these components than their attentional information control (nutrition and lifestyle information) counterparts; (b) those exposed to the threat appraisal information grounded in the PMT components of severity and vulnerability will show lower intentions to vape and lower vaping use compared to their attentional information control counterpart; and (c) increases both severity and vulnerability of vaping usage will be associated with a reduction in intentions to vape. Furthermore, reduction in intentions to vaping will be associated with lower vaping use.

## Method

### Participants and sample size calculation

A convenience sample of Canadian university undergraduate students (*n* = 77) participated in this web-based study. Table 1 contains demographic characteristics of the sample. All participants must have vaped at least three times in the 30 days prior to enrolling in the study for inclusion. As shown in Table 1, the majority of the participants vaped between 5 and 15 days per month (35.65%) and had tried other smoking products/devices (77.55%). The a priori sample size calculation took into account the medium-to-large intention effect size (ηp^2^ = 0.09) obtained by Droulers et al. (2017) and the medium intention effect size (ηp^2^ = 0.05) obtained by Wright et al. (2008) who both used tobacco smoking health information. Based on these results, approximately 20–50 participants were needed per group for a between-group design with an α of 0.05 and a power of 0.80 (Cohen, 1992).

**Table 1.** (Completed data) demographic characteristic for the two treatment conditions.

Variable	PMT (*n =* 41)	Control (*n* = 36)	Statistic (*n* = 77)	*p* Level
Age in years (SD)	21.58 (3.23)	22.69 (3.70)	*F*(7, 49) = 1.69	0.41
Academic year	3.19 (1.35)	3.13 (1.53)	*F*(4, 59) = 0.49	0.74
Gender				
Male	46.3%	44.4%	χ^2^(19, *N* = 83) = 23.59	0.88
Female	53.7%	55.6%		
Other	0.0%	0.0%		
Prefer not to answer	0.0%	0.0%		
Vaping behaviour (past 30 days)				
1–5 days	17.2%	29.2%	χ^2^(76, *N* = 83) = 74.57	0.87
5–15 days	38.0%	33.3%		
16–29 days	17.2%	20.8%		
All 30 days	27.6%	16.7%		
Ethnicity				
Caucasian	61.0%	50.0%	χ^2^(95, *N* = 83) = 108.0	0.42
African American	4.9%	2.7%		
Hispanic American	2.4%	5.6%		
Asian American	12.2%	22.2%		
Indigenous peoples	12.2%	5.6%		
Other	7.3%	13.9%		
Household income				
Under $25,000	17.1%	25.0%	χ^2^(76, *N* = 83) = 83.00	0.41
$25,000–$60,000	19.5%	13.9%		
$60,000–$100,000	26.8%	30.6%		
$100,000–$150,000	22.0%	22.2%		
Prefer not to answer	14.6%	8.3%		
Employment status				
Employed full-time (>40 hours/week)	14.6%	13.9%	χ^2^(57, *N* = 83) = 52.00	0.24
Employed part-time (<40 hours/week)	48.8%	41.7%		
Unemployed	29.3%	33.3%		
Self-employed	7.3%	11.1%		
Age first tried vaping				
10 or younger	0.0%	0.0%	χ^2^(36, *N* = 83) = 34.89	0.17
10–15	14.3%	3.5%		
16–18	42.9%	41.4%		
19 or older	42.9%	55.1%		
Parental vaping presence				
Yes	13.9%	20.0%	χ^2^(19, *N* = 83) = 25.12	0.40
No	86.1%	80.0%		
Four closest friends that vape				
None	8.3%	3.3%	χ^2^(76, *N* = 83) = 91.38	0.84
One	19.4%	40.0%		
Two	24.0%	16.7%		
Three	22.2%	16.7%		
All four	25.0%	23.3%		
Other products/devices used				
Yes	86.1%	69.0%	χ^2^(38, *N* = 83) = 53.59	0.07
No	13.9%	24.1%		
Prefer not to say	0.0%	6.9%		

Standard deviation presented in parentheses.

PMT: protection motivation theory group; *Control* general health information group; *Academic year* within institution.

### Design and procedure

**Figure A1.**
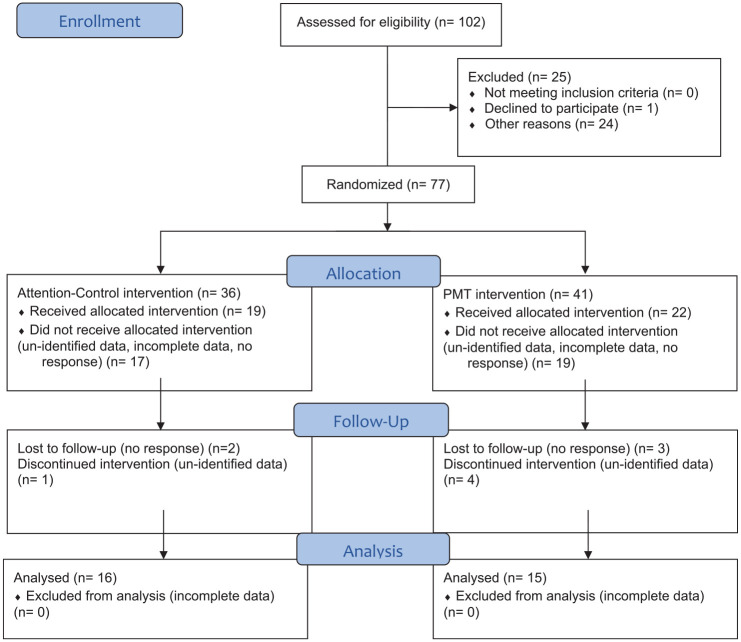
CONSORT 2010 flow diagram. Number of potential participants reached through Facebook advertisements and the Mass Email Recruitment System remains uncertain.

Study procedures were pre-registered with Clinical Trials and adheres to the requirements of the institutional registry. Data were collected in January 2021. Recruitment began in January 2021 and continued until data collection concluded in April 2021, at which point treatment effects on study outcomes were analyzed. Participants were recruited through digital posters in university student Facebook groups and the Mass Email Recruitment system at the institution. Participants were targeted to participate if they self-identified as a regular vaper (vaping at least 3× in the past 30 days) and were 18 years of age or older while being enrolled within an accredited Canadian university. Participants were allocated a participant ID (XX-YYY) upon enrollment in the study. Questionnaires used to collect data were labeled using participants’ ID and no identifiers were associated with participant ID to protect their anonymity. All communication and procedures (consent forms, distributed incentives, intervention videos, and questionnaire links) were conducted through registered emails of each respective participant. Thus, all participants read the Letter of Information and provided informed written consent prior to participation in the study. Participants were blinded and randomly allocated to one of two treatment groups using block randomization and a random number generator to allocate participants using a 1:1 ratio with block sizes of two. Participants in the PMT present group watched an 8-minute informational video that explained the current research and health risks associated with vaping, within the context of a threat appraisal focus. By design, this video focused on the severity of vaping behaviour on health and the susceptibility of young adult populations to adverse health effects with commentary by healthcare professionals and students who previously identified as regular vapers. Participants in the PMT absent group featured an 8-minute nutritional information video as an attention control (See Appendix for details on the development of the PMT and attention control material). Viewing of interactive features were disabled (number of views, comments and “Like/Dislike” buttons were disabled) to prevent participant interaction and video settings were set as “Unlisted” (only those with the video link could open the video). Both groups were sent five anonymous online survey links. Study design followed the Consort checklist of information for reporting randomised trials (see Appendix CONSORT, 2010; Figure A1). VeraCrypt encryption software was used to secure participant information on the SI’s laptop and BitLocker-encryption was used for Personal Vault OneDrive data storage including study data, source data (including surveys), and Letter of Information and Consent. The study’s protocol and materials underwent full board review and were approved by the institutions Health Sciences Research Ethics Board (HSREB) in December 2020.

### Measures

#### Threat appraisal

Perceived vulnerability (PV) perceived severity (PS) were each assessed by four 10-point items (0 = strongly disagree to 10 = strongly agree), derived from past PMT literature (Courneya and Hellsten, 2001). Example items included, “I feel that my chance of developing health problems at some point because of vaping is” (PV) and “I feel that it would be very serious for me to develop health problems if I continue to vape” (PS). The average internal consistency Cronbach’s α for PS over the four time points was α = 0.88, for PV α = 0.92.

#### Goal intention

Goal intention was assessed by three 10-point purpose-built items (0 = *Not At All* to 10 = *Very Seriously*). An example item is, “Would you seriously consider starting a structured program designed to help you reduce or quit vaping to decrease your risk of developing health problems?”. The average internal consistency Cronbach’s α for this intention scale over the four time points was α = 0.92.

#### Behaviour

Vaping behaviour was assessed by one 5-point purpose-built item (0 = *0 times* to 5 = *More Than 30*). The repeated measure item is “During the past X days, how many days did you vape?” (see Appendix for additional measures used).

#### Statistical analyses

All analyses were conducted using IBM SPSS Statistics 25 for MacOS. Presentation of statistical results and analyses methods for both completed data and imputed data (sensitivity) are illustrated separately below. Both data sets used one-way ANOVAs and chi-square procedures to ensure that there were no systematic differences between groups on demographic characteristics. Separate 2 (group) by 4 (time) repeated measures ANOVAs were conducted for each of the variable measures: PV, PS, intention, and behaviour. Pearson correlation analyses were used to measure the statistical strength and direction of relationship between threat appraisal variables, vaping intention, and behaviour. Finally, a linear regression model was conducted to predict the parameters of threat appraisal on intention and intention on behaviour.

## Results (completed data)

### Group equivalency

Table 1 shows the means, standard deviations, and proportions of descriptive characteristics for the total sample. One-way ANOVAs and chi-square analyses showed no significant difference between treatment conditions on any of demographic characteristics recorded. Hence, it was deemed unnecessary to use demographic variables as covariates in the subsequent analyses.

### Group differences

**Figure 1.**
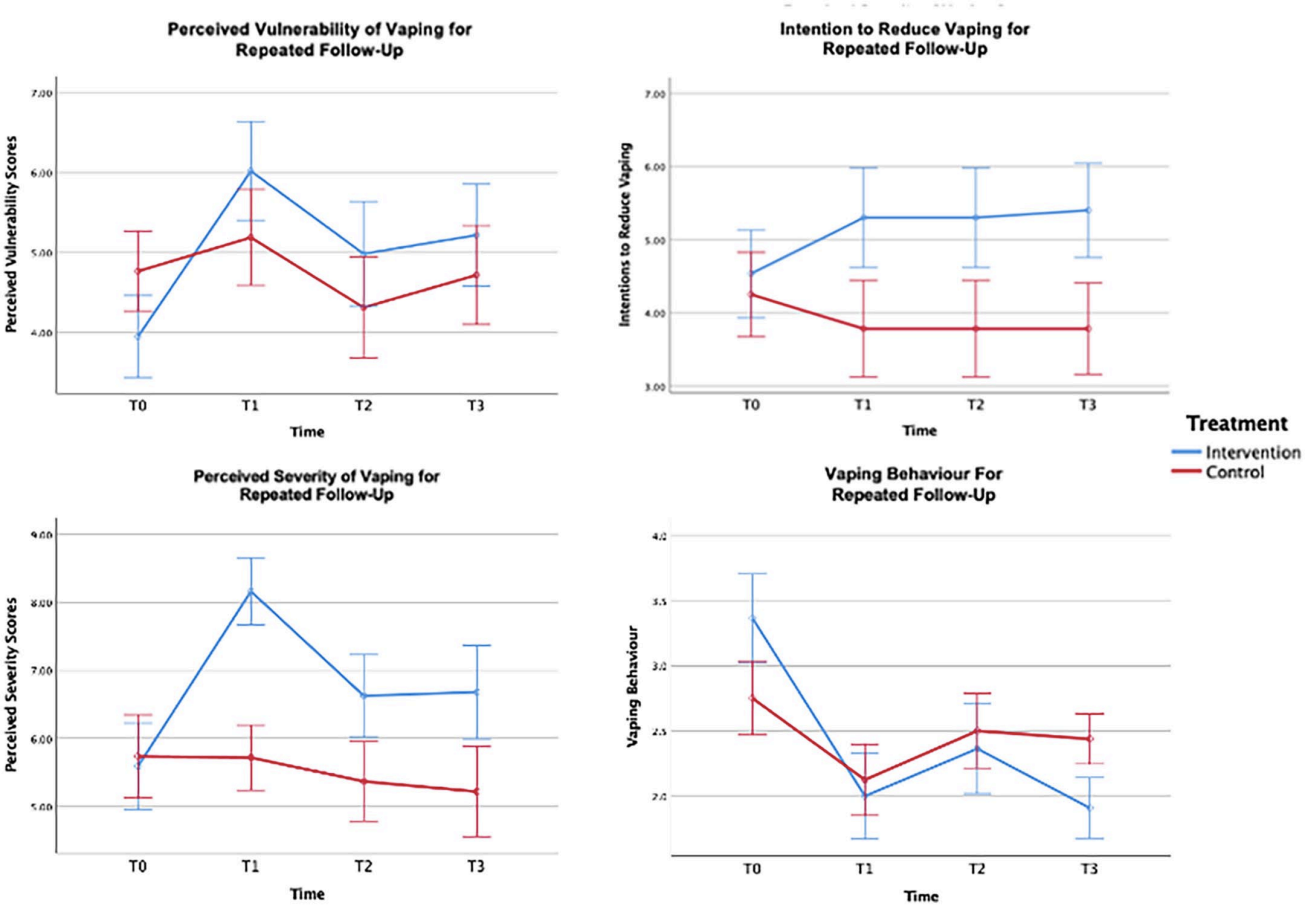
(Complete data) mean and standard error scores between treatment groups across time for PV, PS, intention, and behaviour. T0: Baseline; T1: Day 7; T2: Day 30; T3: Day 45.

Separate one-way factorial repeated measure ANOVAs showed that the two treatment groups differed significantly across time on PS and PV. Specifically, the PMT intervention group scored higher on both threat components than their attention control counterparts. Non-significant treatment by time group differences for intention to reduce vaping behaviour were found. The effect size for this interaction was moderate in size and favoured the PMT intervention group. Non-significant effects were revealed between treatment groups for vaping behaviour for the follow-up assessments. The effect size for this interaction was large, and favoured the PMT intervention group, particularly towards the end of treatment (Figure 1; Supplementary File, Tables 1 and 2).

### Correlation analysis

Bivariate Pearson correlations between threat appraisal variables, vaping intention, and vaping behaviour at baseline and follow-up are presented in Table 2. Perceived vulnerability and severity were significantly related to each other and intention at multiple follow-up time points. Goal intention was not found to be significantly related to behavior at any time point.

**Table 2.** (Completed data) bivariate correlations between the modified PMT variables with intention and behaviour.

Variable	*n*	Mean	SD	1	2	3	4
Baseline (T0)							
1. Perceived vulnerability	63	5.29	2.48	—	0.71**	0.66**	0.28*
2. Perceived severity	62	6.30	2.36		—	0.49**	0.14
3. Intention	64	5.02	2.52			—	0.13
4. Behaviour	65	2.97	1.36				—
Day 7 (T1)							
1. Perceived vulnerability	51	5.89	2.42	—	0.56**	0.59**	−0.03
2. Perceived severity	50	7.16	1.97		—	0.31	−0.09
3. Intention	40	4.70	2.76			—	0.18
4. Behaviour	50	2.32	1.24				—
Day 30 (T2)							
1. Perceived vulnerability	39	4.76	2.38	—	0.72**	0.78**	0.19
2. Perceived severity	39	6.13	2.15		—	0.51**	0.18
3. Intention	38	4.67	2.78			—	0.26
4. Behaviour	38	2.53	1.20				—
Day 45 (T3)							
1. Perceived vulnerability	31	4.96	2.44	—	0.45*	0.38*	0.12
2. Perceived severity	31	5.91	2.58		—	0.53*	0.16
3. Intention	31	4.56	2.59			—	0.23
4. Behaviour	31	2.16	0.82				—

* *p <* 0.05. **p < 0.01.

### Linear regression analysis

Linear regression analysis between bivariate variables (PV, PS, intention) are presented (Table 3). The linear regression for predicting intention found both PV and PS to be significant influencers. Moreover, PV is revealed to be the strongest measure of vaping intention with significant effects at T0, T1, and T2, followed by a significant PS effect at T3.

**Table 3.** (Completed data) linear regression analyses predicting intention.

Variable	Baseline (T0)		Day 7 (T1)		Day 30 (T2)		Day 45 (T3)	
	*B* (SE *B*)	β	*B* (SE *B*)	β	*B* (SE *B*)	β	*B* (SE *B*)	β
Perceived vulnerability	0.31 (0.28)	0.27	0.67 (0.18)***	0.56	1.0 (0.18)***	0.86	0.17 (0.19)	0.16
Perceived severity	0.21 (0.26)	0.22	−0.04 (0.22)	−0.03	−0.12 (0.22)	−0.09	0.41 (0.19)*	0.41

Only PMT variables which were significantly correlated with intention were entered in each regression model.

* *p <* 0.05. **p < 0.01. ***p < 0.001.

### Missing and excluded data

Multiple imputation (MI) analyses were used for all missing data points across all variables within both treatment groups. The MI process included combining five plausible imputed data sets (estimated values on other available information) and appropriately combining results obtained from each of them (Wu et al., 2014). Through data analysis patterns of missing values our missing data was shown as “missing at random,” with 42.24% of all values collected containing missing data, negating the decision to use “single imputation” or “complete case analysis” strategies (Jakobsen et al., 2017).

### Imputed group differences

Separate one-way ANOVAs showed that both treatment groups differed significantly on PS and PV across time. Significant treatment group differences across time for intention to reduce vaping behaviour and vaping use also were found. In all ANOVAS, the PMT intervention group showed higher PS, PV, and intentions as well as lower vaping use compared to the attention control group (see Supplemental File, Figure 1, Tables 4 and 5).

### Imputed correlation analysis

Correlations among the variable of interest can be found in Table 4. Perceived vulnerability and severity were significantly related to each other and intention at multiple follow-up time points, however, only PS showed significant effect on intention and behaviour at final follow-up (T3). In addition, intention did not maintain a consistently significant association with vaping behaviour. Specifically, no association was found at baseline, T1, and T3. However, a positive association was found at T2, suggesting that higher intentions to reduce vaping are associated with higher rates of vaping behaviour. Due to this contradictory finding this relationship was not pursued further through regression analysis.

**Table 4.** (Imputed data) bivariate correlations between the modified PMT variables with intention and behaviour.

Variable	*n*	Mean	SD	1	2	3	4
Baseline (T0)							
1. Perceived vulnerability	448	5.12	2.65	—	0.61**	0.61**	0.25**
2. Perceived severity	447	6.36	2.61		—	0.38**	0.10*
3. Intention	449	4.81	3.05			—	0.08
4. Behaviour	450	2.97	1.35				—
Day 7 (T1)							
1. Perceived vulnerability	436	6.07	4.06	—	0.43**	0.34**	0.03
2. Perceived severity	435	7.31	3.23		—	0.20**	−0.00
3. Intention	425	4.70	2.49			—	0.09
4. Behaviour	435	2.70	1.33				—
Day 30 (T2)							
1. Perceived vulnerability	424	5.54	4.01	—	0.80**	0.43**	0.19**
2. Perceived severity	424	6.71	3.33		—	0.29**	0.19**
3. Intention	423	4.70	2.49			—	0.17**
4. Behaviour	423	2.93	1.39				—
Day 45 (T3)							
1. Perceived vulnerability	416	4.13	20.28	—	−0.20**	0.59**	0.02
2. Perceived severity	416	10.23	18.68		—	0.13**	−0.18**
3. Intention	416	4.94	8.05			—	−0.05
4. Behaviour	416	2.02	0.81				—

* *p* < 0.05. **p < 0.01.

### Imputed linear regression analysis

Linear regression analysis between significant bivariate variables (PV, PS, and intention) are presented in Table 5. Both PV and PS predicted significant amounts of unique variance of intention. PV showed to be the strongest measure of vaping intention with significant effects at all time points, with PS strengthening in effect over time, holding a significant effect at T2 and T3 time points.

**Table 5.** (Imputed data) linear regression analyses predicting intention.

Variable	Baseline (T0)		Day 7 (T1)		Day 30 (T2)		Day 45 (T3)	
	*B* (SE *B*)	β	*B* (SE *B*)	β	*B* (SE *B*)	β	*B* (SE *B*)	β
Perceived vulnerability	0.72 (0.06)***	0.63	0.20 (0.03)***	0.31	0.33 (0.05)***	0.52	0.30 (0.02)***	0.65
Perceived severity	0.00 (0.06)	0.00	0.06 (0.04)	0.07	−0.11 (0.06)*	−0.15	0.12 (0.02)***	0.26

Only PMT variables which were significantly correlated with intention were entered in each regression model.

* *p* *<* 0.05. **p *<* 0.01. ***p *<* 0.001.

## Discussion

The results of the present study support the view that both threat appraisals (PV and PS) grounded in PMT are effective mechanisms to reduce vaping intentions among a sample of Canadian university students who regularly vape. With respect to vaping use, we found inconsistent and thus less convincing evidence for the effectiveness of the PMT intervention. Beyond these general findings, a number of specific issues warrant further commentary.

Analysis with both completed and imputed data found significant differences between PV and PS favouring the experimental group. The effect sizes were large for both threat appraisal constructs when completed data were used (see Supplemental File, Table 1) and medium when imputed data were used (see Supplemental File, Table 4). The emergence of PS being negatively affected by our threat intervention may be explained by the potential health effects of vaping described by health professionals in the experimental group. As a relatively new product in North America, the potential health effects of vaping remain unclear (Olmedo et al., 2018). To those participating in this study, hearing that vaping may cause sudden pneumonia or a pneumothorax (collapsed lung), likely raised participants’ perception of severity in harm related to vaping. With respect to PV, we were somewhat surprised that this construct was also negatively affected by our threat intervention. Previous research has shown that manipulating vulnerability among young people is difficult because they in general feel less vulnerable to health-related problems (Millstein and Halpern-Felsher, 2002).

Turning to intention, analysis within completed data revealed non-significant treatment by time interaction effect. An inspection of the data shows a positive change in intention to reduce vaping use for the PMT intervention group when compared to the attention control group (Figure 1 and Supplementary File, Table 1). The lack of statistically significant change can be explained by low observed power (β = 0.26) resulting from the small sample size. From the imputed data analysis (see Supplemental File, Figure 1 and Table 4), we saw a significant intention by time interaction effect favouring the intervention group along with a large increase in observed power (β = 0.99). A medium effect size was found (completed data: η_p_^2^ = 0.09; imputed data: η_p_^2^ = 0.05) between the two data sets.

With respect to vaping use, we found inconsistent and thus less convincing evidence for the effectiveness of the PMT intervention (see Figure 1 and Table 2). When using the completed data set, a large non-significant treatment by time interaction effect was found for vaping use (η_p_^2^ = 0.22; *p* = 0.12) (See SupplementalFile, Table 1). The observed power for this analysis was low β = 0.47. In contrast, the imputed data analysis, reached statistical significance (*p* = 0.001), with a high observed power (β = 0.99) and medium effect size (η_p_^2^ = 0.06). Overall, participants in the PMT intervention and attention control group both reported their greatest decline in vaping behaviour from baseline to T1. From T1 to T2, both groups demonstrated a modest increase vaping behaviour. From T2 to T3 the experimental group showed another decline in vaping behaviour whereas vaping use remained unchanged for the attention control group (completed data) or declined to a lesser degree than the experimental group (imputed data).

Overall, we found consistent treatment effects for both completed and imputed data sets. Nevertheless, there was a noticeable discrepancy in effect size among the variables of interest between the completed and imputed data sets. We would conclude with completed data that the effects were large (with the exception of intention which was medium) among the variables of interest. In contrast, we would conclude with imputed data that the effects were medium.

With respect to theoretically driven relationships among the variables of interest, we found evidence that increases in the threat components of PV and PS were associated with reductions in intentions to vape. Amongst both data sets, bivariate correlation analysis revealed that PV corroborated strongest with vaping intention overall (Tables 2 and 4). Completed data analysis revealed PV to be the strongest indicator of intention at three time points (T0, T1, and T2), while PS was shown to be the strongest predictor at final follow-up (T3) (Table 2). Imputed data showed that PV and PS maintained a significant effect for intention at all time points with PV holding the strongest effect at each time point (Table 4). Taken together, these correlation findings imply that the vaping intention reduction effects observed favoring the experimental PMT group likely occurred because the PMT intervention was able to successfully manipulate the threat appraisal constructs PV and PS. Limited and inconsistent evidence was found to support relations between vaping intentions and vaping use (behaviour). In turn, because of the moderate-to-large effect in vaping use favoring the experimental group in both completed and imputed data sets, it is concluded that intention was not responsible for behaviour treatment group differences observed. Not unlike numerous other theories, PMT fails to account for behaviour change as successfully as it accounts for intention change irrespective of the population studied (Gaston and Prapavessis, 2009; Webb et al., 2010). This limitation highlights the existence of an “intention-behaviour gap” and elicits that changing complex behaviours such as vaping requires more than the simple formation of positive intentions (Wu et al., 2014). To this end, the inclusion of action and coping planning components, stemming from seminal work on implementation intentions (Jones et al., 2014; Prapavessis et al., 2016) to bridge the intention-behaviour gap, is required.

There are several strengths to the present study. These include a factually based threat appraisal vaping intervention grounded in PMT that is both cost effective and scalable (i.e., online intervention that can be easily implemented in public and private health settings with limited financial or structural obstacles). This study is not without limitations. For instance, although missing data were at random and appropriate imputation procedures (i.e., multiple imputation) were followed, there were large amounts of missing data, particularly from baseline to time 1 (T1). Future studies should consider implementing more user-friendly survey design features (i.e., restricting participants from submitting their questionnaires before answering each question) to mitigate missing data. Considering the medium to large effect sizes found in the present study, future studies should also aim to recruit over 50 participants per group for a between treatment design with an α level of 0.05 and a power of 0.80 (Cohen, 1992). In addition, the COVID-19 pandemic limited our capability to recruit participants in-person. Moreover, our findings are limited to only Canadian college/university students. Our description of a regular vaper is also derived from previous literature (Gambaryan, 2018) however this characterization can be strengthened through replication of people who vape more than the current sample. Another limitation with the present research is it addressed the effect of static threat information on intentions, and in turn behavior. Thus, future intervention research should consider using dynamic rapid gain models of threat perceptions like looming vulnerability (Riskind, 1997). In addition, purpose-built vaping questions adapted from other health-threating behaviours (i.e., tobacco cigarette usage) were used to assess the PMT threat constructs of PS and PV, as well as the intentions to vape less and actual vape use. These vaping questions need further psychometric evaluation in future studies.

## Conclusion

In conclusion, this is the first study to support the view that presenting isolated factual threat appraisal vaping information grounded in a Protection Motivation Theory framework concerning the possible negative health effects of vaping, may be effective at reducing vaping intentions and to a lesser extent vaping behaviour, among Canadian university students who regularly vape. We recommend replication work to confirm whether such message framing interventions can lead to short and long-lasting vaping behaviour change.

## Supplemental Material

sj-docx-10-hpq-10.1177_13591053221144977 – Supplemental material for Using a protection motivation theory framework to reduce vaping intention and behaviour in Canadian university students who regularely vape: A randomized controlled trial Click here for additional data file. Supplemental material, sj-docx-10-hpq-10.1177_13591053221144977 for Using a protection motivation theory framework to reduce vaping intention and behaviour in Canadian university students who regularely vape: A randomized controlled trial by Babac Salmani and Harry Prapavessis in Journal of Health Psychology

sj-docx-8-hpq-10.1177_13591053221144977 – for Using a protection motivation theory framework to reduce vaping intention and behaviour in Canadian university students who regularely vape: A randomized controlled trial Click here for additional data file. sj-docx-8-hpq-10.1177_13591053221144977 for Using a protection motivation theory framework to reduce vaping intention and behaviour in Canadian university students who regularely vape: A randomized controlled trial by Babac Salmani and Harry Prapavessis in Journal of Health Psychology

sj-pdf-9-hpq-10.1177_13591053221144977 – Supplemental material for Using a protection motivation theory framework to reduce vaping intention and behaviour in Canadian university students who regularely vape: A randomized controlled trial Click here for additional data file. Supplemental material, sj-pdf-9-hpq-10.1177_13591053221144977 for Using a protection motivation theory framework to reduce vaping intention and behaviour in Canadian university students who regularely vape: A randomized controlled trial by Babac Salmani and Harry Prapavessis in Journal of Health Psychology

sj-spv-1-hpq-10.1177_13591053221144977 – for Using a protection motivation theory framework to reduce vaping intention and behaviour in Canadian university students who regularely vape: A randomized controlled trial Click here for additional data file. sj-spv-1-hpq-10.1177_13591053221144977 for Using a protection motivation theory framework to reduce vaping intention and behaviour in Canadian university students who regularely vape: A randomized controlled trial by Babac Salmani and Harry Prapavessis in Journal of Health Psychology

sj-spv-2-hpq-10.1177_13591053221144977 – for Using a protection motivation theory framework to reduce vaping intention and behaviour in Canadian university students who regularely vape: A randomized controlled trial Click here for additional data file. sj-spv-2-hpq-10.1177_13591053221144977 for Using a protection motivation theory framework to reduce vaping intention and behaviour in Canadian university students who regularely vape: A randomized controlled trial by Babac Salmani and Harry Prapavessis in Journal of Health Psychology

sj-spv-3-hpq-10.1177_13591053221144977 – for Using a protection motivation theory framework to reduce vaping intention and behaviour in Canadian university students who regularely vape: A randomized controlled trial Click here for additional data file. j-spv-3-hpq-10.1177_13591053221144977 for Using a protection motivation theory framework to reduce vaping intention and behaviour in Canadian university students who regularely vape: A randomized controlled trial by Babac Salmani and Harry Prapavessis in Journal of Health Psychology

sj-spv-4-hpq-10.1177_13591053221144977 – for Using a protection motivation theory framework to reduce vaping intention and behaviour in Canadian university students who regularely vape: A randomized controlled trial Click here for additional data file. sj-spv-4-hpq-10.1177_13591053221144977 for Using a protection motivation theory framework to reduce vaping intention and behaviour in Canadian university students who regularely vape: A randomized controlled trial by Babac Salmani and Harry Prapavessis in Journal of Health Psychology

sj-spv-5-hpq-10.1177_13591053221144977 – for Using a protection motivation theory framework to reduce vaping intention and behaviour in Canadian university students who regularely vape: A randomized controlled trial Click here for additional data file. sj-spv-5-hpq-10.1177_13591053221144977 for Using a protection motivation theory framework to reduce vaping intention and behaviour in Canadian university students who regularely vape: A randomized controlled trial by Babac Salmani and Harry Prapavessis in Journal of Health Psychology

sj-spv-6-hpq-10.1177_13591053221144977 – for Using a protection motivation theory framework to reduce vaping intention and behaviour in Canadian university students who regularely vape: A randomized controlled trial Click here for additional data file. -spv-6-hpq-10.1177_13591053221144977 for Using a protection motivation theory framework to reduce vaping intention and behaviour in Canadian university students who regularely vape: A randomized controlled trial by Babac Salmani and Harry Prapavessis in Journal of Health Psychology

sj-xlsx-7-hpq-10.1177_13591053221144977 – for Using a protection motivation theory framework to reduce vaping intention and behaviour in Canadian university students who regularely vape: A randomized controlled trial Click here for additional data file. sj-xlsx-7-hpq-10.1177_13591053221144977 for Using a protection motivation theory framework to reduce vaping intention and behaviour in Canadian university students who regularely vape: A randomized controlled trial by Babac Salmani and Harry Prapavessis in Journal of Health Psychology

## Appendix

### Development of PMT and other material

#### PMT video

The PMT present group watched an 8-minute informational video (video link: https://www.youtube.com/watch?v=WTKevKge2dg&t=171s) that explained the current research and health risks associated with vaping, within the context of the two major components of PMT threat appraisal (Perceived Vulnerability and Perceived Severity). By design, this video focused on the severity of vaping behaviour on health and the susceptibility of young adult populations to adverse health effects with commentary by healthcare professionals and students who previously identified as regular vapers. During this video intervention, the severity and vulnerability of vaping among young adults, both the short and long-term health effects were presented by two healthcare professionals from the Pediatric Pulmonology department at the NYU Winthrop Hospital and Langone Hassenfeld Children’s Hospital, respectively. In addition, PMT threat components were emphasized through personal experiences led by students at the University of Utah with information regarding our current knowledge of vaping health effects. The second half of the video included narration by ‘Science Insider’, explaining the negative health impacts of vaping and the lack of research and information that currently exists on popular vaping products, including their potentially devastating impact on the health of young adult populations.

#### Attention control video

The PMT absent group featured an 8-minute nutritional information video (video link: https://www.youtube.com/watch?v=W4RKlLJU8RM) as an attention control strategy titled “Health Effects.” During this video intervention, the general risks and benefits of nutrition and a healthy lifestyle were presented by the Alliance for Aging Research, including an immersive video design, explaining the impact that nutrition may have as we age, reviewed by a Senior Nutritionist followed with narration by a constituent of TED-Ed. The focus of this video was on how a balanced diet and proper lifestyle choices (i.e., adequate sleep, diet, etc.) can benefit your life. In the nutrition and lifestyle choices intervention, the topic of substance abuse was discussed briefly but without depth to illustrate the risks to the health of young adults titled. The nutrition and lifestyle information design was administered as a contact control because it provides informative lifestyle choices regarding nutrition that can help promote overall health without having an underlying link to vaping behaviour and its underlying effect on the status of health. The reasoning behind this is to examine the influence of specific vs. non-specific treatment on variables of interest.

### Measures

#### Demographic and modified youth vaping

Two seven-item vaping Canadian student vaping demographic and tobacco use questionnaires derived from an existing Canadian Student Tobacco, Alcohol, and Drugs Survey (CSTADS) conducted by a consortium of researchers across Canada, centralized at the University of Waterloo (2014). Excluding drugs assessment questions, the Demographic and Youth Vaping Questionnaires collected descriptive information, social influence, current behaviour, and use of past tobacco-based products. Example items include “What is your ethnicity?” (Demographic) and “Have you ever used chewing tobacco, cigarettes, cigars, cigarillos, or little cigars?” (YVQ-A).
