# Supplementary material for: Using a protection motivation theory framework to reduce vaping intention and behaviour in Canadian university students who regularely vape: A randomized controlled trial
Source: J Health Psychol. 2023 Jan 12;28(9):832–45. doi: 10.1177/13591053221144977 (PMC10387725; doi:10.1177/13591053221144977)
Supplement: sj-docx-10-hpq-10.1177_13591053221144977 – Supplemental material for Using a protection motivation theory framework to reduce vaping intention and behaviour in Canadian university students who regularely vape: A randomized controlled trial [file sj-docx-10-hpq-10.1177_13591053221144977.docx]

**Supplementary File**

**
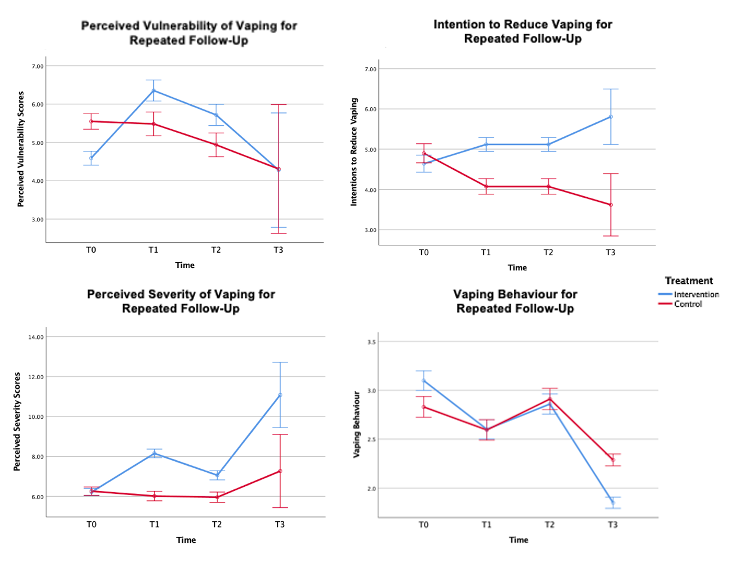
**

**Figure 1 (**Imputed data) mean and standard error scores between treatment groups across time for PV, PS, intention and behaviour

Note. T0 = Baseline, T1 = Day 7, T2 = Day 30, T3 = Day 45

Note. T0 = Baseline, T1 = Day 7, T2 = Day 30, T3 = Day 45

| Variables | *F* (1, 28) | *p* | Partial Eta Squared (ηp2) | Observed Power |
| --- | --- | --- | --- | --- |
| Perceived Vulnerability | 3.28 | .036 | .27 | .69 |
| Perceived Severity | 3.69 | .025 | .31 | .74 |
| Intention | 124.7 | .284 | .09 | .26 |
| Behaviour | 8.08 | .123 | .22 | .47 |

**Table 1** (Completed data) treatment by time interaction effects for threat appraisal, intention, and behaviour between treatment conditions

| PMT Condition  *n* = 15 | T0 (M(SD)) | T1 | T2 | T3 |
| --- | --- | --- | --- | --- |
| Perceived Vulnerability | 3.95 (2.4) | 6.02 (2.4) | 4.98 (2.8) | 5.22 (2.7) |
| Perceived Severity | 5.59 (2.8) | 8.16 (1.5) | 6.62 (2.3) | 6.68 (2.7) |
| Intention | 4.53 (2.5) | 5.30 (2.8) | 5.30 (2.8) | 5.40 (3.0) |
| Behaviour | 3.36 (1.3) | 2.0 (1.2) | 2.36 (1.1) | 1.91 (0.8) |

**Table 2** (Completed data) mean and standard deviation for threat appraisal, intention, and behaviour for PMT and Control condition

| Control Condition  *n* = 16 | T0 (M(SD)) | T1 | T2 | T3 |
| --- | --- | --- | --- | --- |
| Perceived Vulnerability | 4.77 (1.5) | 5.19 (2.4) | 4.31 (2.3) | 4.72 (2.2) |
| Perceived Severity | 5.73 (1.9) | 5.72 (2.1) | 5.37 (2.2) | 5.22 (2.4) |
| Intention | 4.25 (2.2) | 3.78 (2.5) | 3.78 (2.5) | 3.78 (1.9) |
| Behaviour | 2.75 (1.0) | 2.13 (1.0) | 2.50 (1.2) | 2.44 (.73) |

Note. T0 = Baseline, T1 = Day 7, T2 = Day 30, T3 = Day 45

**Table 3** (Imputed data) demographic characteristic for the two treatment conditions

| Variable | PMT (*n =* 82) | Control (*n* = 57) | Statistic (*n* = 139) | *p* level |
| --- | --- | --- | --- | --- |
| Age in years (SD) | 21.91 (3.51) | 22.54 (3.51) | *F*(1, 442) = 3.67 | .55 |
| Academic Year | 3.19 (1.34) | 3.08 (1.56) | *F*(1, 442) = .70 | .73 |
| Gender |  |  |  |  |
| Male | 46.1 % | 45.5 % | χ^2^(1, *N* = 448) = .05 | .94 |
| Female | 53.9 % | 54.5 % |  |  |
| Other | 0.0 % | 0.0 % |  |  |
| Prefer not to answer | 0.0 % | 0.0 % |  |  |
| Vaping Behaviour (Past 30 Days) |  |  |  |  |
| 1-5 days | 17.59 % | 31.01 % | χ^2^(4, *N* = 448) = 15.55 | .61 |
| 5-15 days | 35.71 % | 32.55 % |  |  |
| 16-29 days | 18.68 % | 20.94 % |  |  |
| All 30 days | 28.02 % | 15.50 % |  |  |
| Ethnicity |  |  |  |  |
| Caucasian | 60.1 % | 50.0 % | χ^2^(5, *N* = 448) = 17.75 | .30 |
| African American | 4.4 % | 2.6 % |  |  |
| Hispanic American | 2.6 % | 5.8 % |  |  |
| Asian American | 15.3 % | 19.9 % |  |  |
| Indigenous Peoples | 11.0 % | 6.4 % |  |  |
| Other | 6.6 % | 15.3 % |  |  |
| Household Income |  |  |  |  |
| Under $25,000 | 17.1% | 25.7 % | χ^2^(4, *N* = 448) = 9.06 | .44 |
| $25,000-$60,000 | 17.5 % | 16.0 % |  |  |
| $60,000-$100,000 | 30.7 % | 26.9 % |  |  |
| $100,000-$150,000 | 20.6 % | 23.7 % |  |  |
| Prefer not to answer | 14.1 % | 7.7 % |  |  |
| Employment Status |  |  |  |  |
| Employed full-time (>40 hrs/wk) | 16.2 % | 10.9 % | χ^2^(3, *N* = 448) = 6.3 | .56 |
| Employed part-time (<40 hrs/wk) | 46.5 % | 42.3 % |  |  |
| Unemployed | 27.2 % | 37.2 % |  |  |
| Self-employed | 10.1 % | 9.6 % |  |  |
| Age First Tried Vaping |  |  |  |  |
| 10 or younger | 0.0 % | 0.0 % | χ^2^(2, *N* = 382) = 13.21 | .18 |
| 10-15 | 13.6 % | 3.7 % |  |  |
| 16-18 | 43.2 % | 40.7 % |  |  |
| 19 or older | 43.2 % | 55.6 % |  |  |
| Parental Vaping Presence |  |  |  |  |
| Yes | 13.2 % | 21.0 % | χ^2^(1, *N* = 393) = 4.72 | .38 |
| No | 86.8 % | 79.0 % |  |  |
| Four Closest Friends that Vape |  |  |  |  |
| None | 8.9 % | 2.2 % | χ^2^(4, *N* = 393) = 27.00 | .85 |
| One | 20.5 % | 39.1 % |  |  |
| Two | 25.8 % | 15.2 % |  |  |
| Three | 21.1 % | 18.1 % |  |  |
| All four | 23.7 % | 25.4 % |  |  |
| Other Products/ Devices Used |  |  |  |  |
| Yes | 84.4 % | 70.1 % | χ^2^(2, *N* = 388) = 21.01 | .10 |
| No | 15.6 % | 22.6 % |  |  |
| Prefer not to say | 0.0 % | 7.3 % |  |  |

*Note.* Standard deviation presented in parentheses; *PMT* protection motivation theory group, *Control* general health information group, *Academic year* within institution

**Table 4** (Imputed data) treatment by time interaction effects for threat appraisal, intention, and behaviour between treatment conditions

| Variables | *F* (3, 413) | *p* | Partial Eta Squared (ηp2) | Observed Power |
| --- | --- | --- | --- | --- |
| Perceived Vulnerability | 8.52 | .001 | .06 | .99 |
| Perceived Severity | 10.08 | .001 | .07 | 1.0 |
| Intention | 815.6 | .001 | .05 | .99 |
| Behaviour | 68.04 | .001 | .06 | .99 |

**Table 5** (Imputed data) mean and standard deviation for threat appraisal, intention, and behaviour for PMT and Control conditions

| PMT Condition  *n* = 244 | T0 (M(SD)) | T1 | T2 | T3 |
| --- | --- | --- | --- | --- |
| Perceived Vulnerability | 4.71 (2.8) | 6.42 (3.9) | 5.86 (4.0) | 4.09 (21.7) |
| Perceived Severity | 6.34 (2.8) | 8.15 (3.0) | 7.14 (3.2) | 11.96 (19.9) |
| Intention | 4.66 (2.9) | 5.08 (2.5) | 5.08 (2.5) | 5.89 (9.1) |
| Behaviour | 3.06 (1.4) | 2.69 (1.4) | 2.93 (1.4) | 1.84 (0.8) |

| Control Condition  *n* = 172 | T0 (M(SD)) | T1 | T2 | T3 |
| --- | --- | --- | --- | --- |
| Perceived Vulnerability | 5.71 (2.4) | 5.54 (4.1) | 5.06 (3.8) | 4.23 (17.0) |
| Perceived Severity | 6.37 (2.3) | 6.08 (3.1) | 6.08 (3.6) | 7.67 (16.1) |
| Intention | 5.03 (3.2) | 4.13 (2.4) | 4.13 (2.4) | 3.59 (5.9) |
| Behaviour | 2.84 (1.2) | 2.64 (1.3) | 2.95 (1.4) | 2.27 (0.8) |

Note. T0 = Baseline, T1 = Day 7, T2 = Day 30, T3 = Day 45

| Section/ Topic | Item No | CONSORT 2010 checklist of information to include when reporting a randomised trial*  Checklist item | Reported on page No |
| --- | --- | --- | --- |
| Title and abstract | | | |
|  | 1a | Identification as a randomised trial in the title | **1** |
|  | 1b | Structured summary of trial design, methods, results, and conclusions (for specific guidance see CONSORT for abstracts) | **2** |
| Introduction | | | |
| Background and objectives | 2a | Scientific background and explanation of rationale | **2** |
|  | 2b | Specific objectives or hypotheses | **3** |
| Methods | | | |
| Trial design | 3a | Description of trial design (such as parallel, factorial) including allocation ratio | **4** |
|  | 3b | Important changes to methods after trial commencement (such as eligibility criteria), with reasons | **n/a** |
| Participants | 4a | Eligibility criteria for participants | **4** |
|  | 4b | Settings and locations where the data were collected | **4** |
| Interventions | 5 | The interventions for each group with sufficient details to allow replication, including how and when they were actually administered | **11** |
| Outcomes | 6a | Completely defined pre-specified primary and secondary outcome measures, including how and when they were assessed | **2** |
|  | 6b | Any changes to trial outcomes after the trial commenced, with reasons | **n/a** |
| Sample size | 7a | How sample size was determined | **4** |
|  | 7b | When applicable, explanation of any interim analyses and stopping guidelines | **7** |
| Randomisation: |  |  |  |
| Sequence generation | 8a | Method used to generate the random allocation sequence | **4** |
|  | 8b | Type of randomisation; details of any restriction (such as blocking and block size) | **4** |
| Allocation concealment mechanism | 9 | Mechanism used to implement the random allocation sequence (such as sequentially numbered containers), describing any steps taken to conceal the sequence until interventions were assigned | **4** |
| Implementation | 10 | Who generated the random allocation sequence, who enrolled participants, and who assigned participants to interventions | **4** |
| Blinding | 11a | If done, who was blinded after assignment to interventions (for example, participants, care providers, those assessing outcomes) and how | **4** |
|  | 11b | If relevant, description of the similarity of interventions | **11** |
| Statistical methods | 12a | Statistical methods used to compare groups for primary and secondary outcomes | **4** |
|  | 12b | Methods for additional analyses, such as subgroup analyses and adjusted analyses | **n/a** |
| Results | | | |
| Participant flow (a diagram is strongly recommended) | 13a | For each group, the numbers of participants who were randomly assigned, received intended treatment, and were analysed for the primary outcome | **11** |
|  | 13b | For each group, losses and exclusions after randomisation, together with reasons | **11** |
| Recruitment | 14a | Dates defining the periods of recruitment and follow-up | **4** |
|  | 14b | Why the trial ended or was stopped | **n/a** |
| Baseline data | 15 | A table showing baseline demographic and clinical characteristics for each group | **5** |
| Numbers analysed | 16 | For each group, number of participants (denominator) included in each analysis and whether the analysis was by original assigned groups | **11** |
| Outcomes and estimation | 17a | For each primary and secondary outcome, results for each group, and the estimated effect size and its precision (such as 95% confidence interval) | **8** |
|  | 17b | For binary outcomes, presentation of both absolute and relative effect sizes is recommended | **n/a** |
| Ancillary analyses | 18 | Results of any other analyses performed, including subgroup analyses and adjusted analyses, distinguishing pre-specified from exploratory | **8** |
| Harms | 19 | All important harms or unintended effects in each group (for specific guidance see CONSORT for harms) | **n/a** |
| Discussion | | | |
| Limitations | 20 | Trial limitations, addressing sources of potential bias, imprecision, and, if relevant, multiplicity of analyses | **9** |
| Generalisability | 21 | Generalisability (external validity, applicability) of the trial findings | **9** |
| Interpretation | 22 | Interpretation consistent with results, balancing benefits and harms, and considering other relevant evidence | **9** |
| Other information | | |  |
| Registration | 23 | Registration number and name of trial registry | **4** |
| Protocol | 24 | Where the full trial protocol can be accessed, if available | **4** |
| Funding | 25 | Sources of funding and other support (such as supply of drugs), role of funders | **12** |
