# Supplementary material for: Using a protection motivation theory framework to reduce vaping intention and behaviour in Canadian university students who regularely vape: A randomized controlled trial
Source: J Health Psychol. 2023 Jan 12;28(9):832–45. doi: 10.1177/13591053221144977 (PMC10387725; doi:10.1177/13591053221144977)
Supplement: sj-docx-8-hpq-10.1177_13591053221144977 – for Using a protection motivation theory framework to reduce vaping intention and behaviour in Canadian university students who regularely vape: A randomized controlled trial [file sj-docx-8-hpq-10.1177_13591053221144977.docx]

**Explanatory Memo**

All analyses were conducted using IBM SPSS Statistics 25 for MacOS. The current article includes the complete raw dataset collected in the study including the participants’ dataset, syntax file and log files for analysis. Pending acceptance for publication, all of the data files will be automatically uploaded to the Figshare repository. All analyses were by intention-to-treat and included all participants. Missing values (T0, T1...) were replaced using a multiple imputation analyses methodology and computed separately from completed data analyses (Jakobsen et al., 2017). Presentation of statistical results and analyses methods for both completed data and sensitivity data (imputed) are illustrated separately. Both data sets used one-way ANOVAs and chi-square procedures to ensure that there were no systematic differences between groups on demographic characteristics. Separate 2 (group) by 4 (time) repeated measures ANOVAs were conducted for each of the variable measures: PV, PS, intention, and behaviour. Pearson correlation analyses were used to measure the statistical strength and direction of relationship between threat appraisal variables and vaping intention and behaviour based on the method of covariance. Finally, a linear regression model was conducted to predict the parameters of threat appraisal on intention and intention on behaviour variables.
