# Supplementary material for: Using a protection motivation theory framework to reduce vaping intention and behaviour in Canadian university students who regularely vape: A randomized controlled trial
Source: J Health Psychol. 2023 Jan 12;28(9):832–45. doi: 10.1177/13591053221144977 (PMC10387725; doi:10.1177/13591053221144977)
Supplement: sj-pdf-9-hpq-10.1177_13591053221144977 – Supplemental material for Using a protection motivation theory framework to reduce vaping intention and behaviour in Canadian university students who regularely vape: A randomized controlled trial [file sj-pdf-9-hpq-10.1177_13591053221144977.pdf]

Separate Readme PDF

Included Files:

1. Title page (.docx)
   a. Cover page including title for main document submission
2. Main document (.docx)
   a. Manuscript
3. Supplementary file (.docx)
   a. Tables and figures not included in the main document but available for Journal readers to enhance their understanding of findings
4. Figures and other files created outside Word (.docx)
   a. Figure created outside of Word using SPSS
5. Explanatory memo (.docx)
   a. Explanation of software used for data analysis and instruction on how a replication can be conducted
6. Full data set (.xlsx)
   a. Full set of raw data for open access to the readers
7. Intention and behavour ANOVA (.spv)
   a. Intention and behaviour analysis of variance over time (repeated measures (4))
8. General linear model (.spv)
   a. PMT variable analysis of variance over time (repeated measures (4))
9. Linear regression (.spv)
   a. Data analysis output for PV, PS, Intention and Behaviour variables
10. Pearson correlation (.spv)
    a. Data analysis output for PV, PS, Intention and Behaviour variables
11. Imputed intention and behaviour ANOVA (.spv)
    a. Data analysis output for imputed intention and behaviour over time (repeated measures (4))
12. Multiple imputed data analyses results (.spv)
    a. Data analysis output for imputed data (repeated measures (4))
13. Separate Readme PDF (.spv)
    a. Listing of included documents and instructions on how a replication can be conducted

Replication instruction

Participants were recruited on a concurrent basis through poster advertisements on university student Facebook groups and the Mass Email Recruitment system at Western. Individuals that self-identified as eligible emailed the student investigator (SI) where the Letter of Information and Consent was relayed back to the prospective email contact of the individual. Eligible participants that signed the consent form then emailed the forms back to the SI. Participants were allocated to one of two experimental groups using a blocked randomization method; participants were recruited on a concurrent basis and randomized within blocks such that an equal number are assigned to each treatment. To avoid the presence of stratification errors we reviewed our allocation design before administering study intervention and purpose- questionnaires to prevent

participant mismanagement during the protocol (no participant or researcher blinding was present).

The baseline assessment was comprised of identifiable questionnaires to assess their history and experience with vaping and measure their intention to vape less, incorporated within the 4-questionnaire links: Demographic Assessment, Youth Vaping, and PMT (I & II). At Day 7 (T1), participants were emailed their respective video link along with the questionnaires and were instructed to complete the surveys after watching their videos. The study intervention was a single site trial delivered as a video link to the email provided by the participant; both intervention videos were played on YouTube and participants were instructed to complete the surveys immediately after watching the video attached to the email sent to them. The participants completed self-reported questionnaires at 3 follow-up periods after baseline in the 6-week protocol (all questionnaires were sent by the SI to the email provided by the participants). As illustrated in Schema 3.3, self-reported vaping behaviour questionnaires were managed at Baseline, Day 7 (T1), Day 30 (T2), and Day 45 (T3). For every questionnaire set date, the participants had 7 days to complete that compound of questionnaires. A follow-up "reminder" email was sent by the SI to the participant emails of those who had failed to submit that set of questionnaires. The follow-up emails were designed to remind the participants that if they failed to submit the questionnaires within the following 3 days, they would be withdrawn from the study. All questionnaire links were created using the Qualtrics Survey Software and were distributed by the SI to the email provided by the individual participants.

Participants were allocated a participant ID (XX-YYY) upon enrollment in the study. Questionnaires used to collect data were labeled using participants' ID and no identifiers were associated with participant ID to protect their anonymity. VeraCrypt encryption software was used to secure participant information on the SI's laptop and BitLocker-encryption was used for Personal Vault OneDrive data storage including study data, source data (including surveys), and Letter of Information and Consent.

All analyses were conducted using IBM SPSS Statistics 25 for MacOS. All analyses were by intention-to-treat and included all participants. Missing values (T0, T1...) were replaced using a multiple imputation analyses methodology and computed separately from completed data analyses (Jakobsen et al., 2017). Presentation of statistical results and analyses methods for both completed data and sensitivity data (imputed) are illustrated separately. Both data sets used one-way ANOVAs and chi-square procedures to ensure that there were no systematic differences between groups on demographic characteristics. Separate 2 (group) by 4 (time) repeated measures ANOVAs were conducted for each of the variable measures: PV, PS, intention, and behaviour. Pearson correlation analyses were used to measure the statistical strength and direction of relationship between threat appraisal variables and vaping intention and behaviour based on the method of covariance. Finally, a linear regression model was conducted to predict the parameters of threat appraisal on intention and intention on behaviour variables.
